# Robust spike-specific CD4^+^ and CD8^+^ T cell responses in SARS-CoV-2 vaccinated hematopoietic cell transplantation recipients: a prospective, cohort study

**DOI:** 10.3389/fimmu.2023.1210899

**Published:** 2023-07-07

**Authors:** Lorenzo Federico, Tor Henrik Anderson Tvedt, Murat Gainullin, Julie Røkke Osen, Viktoriia Chaban, Katrine Persgård Lund, Lisa Tietze, Trung The Tran, Fridtjof Lund-Johansen, Hassen Kared, Andreas Lind, John Torgils Vaage, Richard Stratford, Simen Tennøe, Brandon Malone, Trevor Clancy, Anders Eivind Leren Myhre, Tobias Gedde-Dahl, Ludvig André Munthe

**Affiliations:** ^1^ Department of Immunology, Oslo University Hospital, Oslo, Norway; ^2^ KG Jebsen Centre for B Cell Malignancies, Institute of Clinical Medicine, University of Oslo, Oslo, Norway; ^3^ Department of Haematology, Oslo University Hospital, Oslo, Norway; ^4^ ImmunoLingo Convergence Center, Institute of Clinical Medicine, University of Oslo, Oslo, Norway; ^5^ Department of Microbiology, Oslo University Hospital, Oslo, Norway; ^6^ Institute of Clinical Medicine, University of Oslo, Oslo, Norway; ^7^ NEC OncoImmunity AS, Oslo, Norway

**Keywords:** COVID-19, HSCT = hematopoietic stem cell transplant, CD8 lymphocytes +, CD4 lymphocyte +, vaccine, SARS -CoV -2

## Abstract

Poor overall survival of hematopoietic stem cell transplantation (HSCT) recipients who developed COVID-19 underlies the importance of SARS-CoV-2 vaccination. Previous studies of vaccine efficacy have reported weak humoral responses but conflicting results on T cell immunity. Here, we have examined the relationship between humoral and T cell response in 48 HSCT recipients who received two doses of Moderna’s mRNA-1273 or Pfizer/BioNTech’s BNT162b2 vaccines. Nearly all HSCT patients had robust T cell immunity regardless of protective humoral responses, with 18/48 (37%, IQR 8.679-5601 BAU/mL) displaying protective IgG anti-receptor binding domain (RBD) levels (>2000 BAU/mL). Flow cytometry analysis of activation induced markers (AIMs) revealed that 90% and 74% of HSCT patients showed reactivity towards immunodominant spike peptides in CD8^+^ and CD4^+^ T cells, respectively. The response rate increased to 90% for CD4^+^ T cells as well when we challenged the cells with a complete set of overlapping peptides spanning the entire spike protein. T cell response was detectable as early as 3 months after transplant, but only CD4^+^ T cell reactivity correlated with IgG anti-RBD level and time after transplantation. Boosting increased seroconversion rate, while only one patient developed COVID-19 requiring hospitalization. Our data suggest that HSCT recipients with poor serological responses were protected from severe COVID-19 by vaccine-induced T cell responses.

## 1 Introduction

Haematopoietic stem cell transplantation (HSCT) is increasingly performed each year on patients with various conditions, such as haematological cancers and immune system deficiencies (1). Patients who underwent HSCT and contracted COVID-19 before the availability of SARS-CoV-2 vaccines had mortality rates that exceeded 20% (2, 3). Furthermore, studies have shown that HSCT patients had poor serological responses to (mRNA) SARS-CoV-2 vaccination (4), and a quarter of patients lacked anti-RBD spike IgG response after two doses (5–8). Response to vaccination in HSCT recipients is impaired during the early posttransplant period but improves over time. For instance, protective IgG levels after influenza vaccine can be detected two years following HSCT, despite low responsiveness during the first six months post-transplant (9–11). Similarly, the response to childhood immunization vaccines including DTaP, Haemophilus influenzae, poliovirus, pneumococcus, and hepatitis B in HSCT recipients is often inadequate and a patient-tailored vaccination schedule is often required to achieve sufficient efficacy (12). However, patients with graft-versus-host disease (GvHD) who require immunosuppressive treatment exhibit impaired vaccine response for a prolonged period (13).

Given the reduced serological responses to SARS-CoV-2 in vaccinated patients, but the low incidence of severe COVID-19 cases and death (5–8), we hypothesized that vaccine-induced T cell responses may be key to protection. Indeed, HSCT patients who received the influenza-A vaccine develop specific Th cell immunity regardless of the amount of time passed after transplantation or the IgG response (14). Moreover, T cell response is essential for rapid and efficient resolution of COVID-19 (15, 16), protection against severe infection in settings of low antibody levels (17), rapid viral control in subclinical infections (18), and immune responses towards SARS-CoV-2 variants of concern (VOC) with RBD mutations that evade neutralizing antibodies (19).

Despite the significance of cell-mediated immunity in viral infection, the current understanding of T cell responses to SARS-CoV-2 vaccine in HSCT recipients remains incomplete. Some studies detected T cell activation in most vaccinated patients who received transplants a median of 55 months before vaccination (20), and found that the majority of humoral responders also had CD4^+^ T cell responses (21), but other studies reported weak T cell responses of low frequency (22, 23), or failed to evaluate the relative contribution of CD4^+^ and CD8^+^ T cells (24).

We here aimed to define IgG anti-RBD antibody levels and spike-specific CD4^+^ and CD8^+^ T cell immunity as a function of therapy and time from transplant in a cohort of allotransplanted HSCT recipients who received two doses of Moderna/mRNA-1273 or Pfizer/BioNTech BNT162b2. We found that 58% of patients seroconverted but that only 37% had protective anti-RBD IgG antibody levels. Quantification of T cell response by activation induced markers (AIMs) revealed that reactivity of spike-specific CD4^+^ but not CD8^+^ T cells correlated with IgG anti-RBD antibody response and the time elapsed from transplantation. Since T cell immunity was detectable in both T cell subsets as early as 3 months after transplantation and only one patient was hospitalized after Delta VOC infection for severe COVID-19 requiring mechanical ventilation, we conclude that vaccine-induced T cell-mediated immunity protected HSCT patients from severe COVID-19 infection.

## 2 Materials and methods

### 2.1 Patients and study design

Blood and serum samples were collected from 48 HSCT patients and controls prospectively enrolled between March 2021 and February 2022 to examine the immune response following two doses of (mRNA) vaccines Moderna/mRNA-1273 or Pfizer/BioNTech’s BNT162b2. All HSCT patients were negative for IgG anti-RBD Nabs (BAU/mL) before vaccination. HSCT patients had all received allotransplants and 26 were recruited from the graft v host disease (GvHD) clinic where patients that develop GvHD receive prolonged immunosuppression. This group was expected to have weaker vaccine responses (see **Supplementary Table S1**).

*Ex vivo* vaccine-dependent T cell reactivity of HSCT patients was compared to the reactivity of T cells from 18 healthy controls (14 females and 4 males) who had no history of COVID-19 infection and were all negative for IgG anti-nucleocapsid. The median time of blood collection after the last dose was 6 weeks. All procedures followed here were carried on in accordance with institutional and national ethical standards of the responsible committee on human experimentation and with the 2013 Helsinki Declaration. Informed consent was obtained from all study participants and approved by the Health Region South-East Regional Ethics committee.

### 2.2 Reagents

Spike-C (PepTivator® SARS-CoV-2 Prot_S Complete; Miltenyi, # 130-127-953), and Spike-I (PepTivator® SARS-CoV-2 Prot_S; Militenyi, # 130-126-700) pools are collections of lyophilized 15-mers peptides with 11 aa overlaps spanning either the immunodominant regions (Spike-I pool sequence domains: aa 304-338, 421-475, 492-519, 683-707, 741-770, 785-802, and 885-1273), or the entire length (Spike-C pool sequence: aa 5-1273) of the SARS-CoV-2 Spike glycoprotein (Protein QHD43416.1, GenBank MN908947.3). NOI pools are collections of 9 to 10-mers peptides identified by the NEC Immune Profiler algorithm (25). The pools include the following peptides: AHFPREGVF, ASFSTFKCY, DKVFRSSVL, ESIVRFPNI, FAMQMAYRF, FKNLREFVF, FVFKNIDGY, FVSNGTHWFV, GTHWFVTQR, GAAAYYVGY, IPFAMQMAY, IPTNFTISV, KTSVDCTMY, KVGGNYNYLY, LPFNDGVYF, LPIGINITRF, LPPAYTNSF, NSFTRGVYY, RGWIFGTTL, RLITGRLQSL, RAAEIRASA, SIIAYTMSL, SPRRARSVA, TPINLVRDL, TRFASVYAW, TRFQTLLAL, WTAGAAAYY, YLQPRTFLL, YQPYRVVVL, YTNSFTRGVY. Peptides were synthesized by GenScript (Piscataway, NJ, USA) at a purity ≥85%, and pooled at a final concentration of 1.5mg/mL each.

### 2.3 PBMC isolation

PBMC were isolated from whole blood using either CPT tubes (BD vacutainer, # 362782) or Lymphoprep™ (Serumwerk Berburg, # 1858). Blood in CPT tubes was first centrifuged at 1600g for 25 minutes at room temperature and plasma collected and stored at -20°C. PBMC were then transferred into a 50mL tube, washed in cold PBS (Gibco, # 10010-015), and counted. Lymphoprep™ isolation was performed per manufactured protocol. Briefly, blood was pipetted into a 50mL tube, mixed 1:1 at room temperature with PBS, and layered onto 10 to 15mL of Lymphoprep™ in a 50mL falcon tube. Tubes were centrifuged at 800g for 25 minutes and PBMC collected, washed 3 times with PBS (400g, 7 min, 4°C) and resuspended in FBS (Gibco, # 10270-106) complemented with 10% DMSO. After overnight pre-chilling at -80°C in Mr. Frosty (Nalgene™, # 5100-0001) cells were transferred in liquid nitrogen for long-term storage.

### 2.4 Serum measurements

Whole blood was collected in 5mL Vacuette® tubes with serum separator clot activator (Vacuette, # 456073R). Serum was obtained by centrifugation at 2000g and stored at -20°C. Semiquantitative measurement of antibodies to full-length spike protein (Spike-FL) and the receptor-binding domain (RBD) from SARS-CoV-2 was performed using a multiplexed bead-based assay (26). Polymer beads with fluorescent barcodes were coupled successively to neutravidin (Thermo Fisher) and biotinylated viral antigens to generate bead-based protein arrays. Sera were diluted 1: 100 in assay buffer (PBS 1% Tween 20, 10μg/mL D-biotin, 10μg/mL Neutravidin, 0.1% Sodium Azide). Diluted sera were incubated with bead-based arrays in 384 well plates for 30 minutes at 22°C under constant agitation, washed three times in PBS 1% Tween 20 (PBT) and labelled with R-Phycoerythrin (R-PE)-conjugated goat-anti-human IgG (Jackson Immunoresearch). For measurement of neutralizing antibodies, the beads were pelleted after incubation with serum and labelled successively with digoxigenin-conjugated human ACE2 and mouse monoclonal anti-dixogigenin (Jackson Immunoresearch), which was conjugated in-house to R-PE. The beads were analyzed with an Attune NxT flow cytometer (Thermo Fisher), and raw data (fcs.3.1) were analyzed in WinList 3D (Verity Softwarehouse). The median R-PE fluorescence intensity (MFI) of each bead subset was exported to Excel. The MFI of beads coupled with viral antigens was divided by that measured on beads coupled with neutravidin only (relative MFI, rMFI). A total of 979 prepandemic sera and 810 sera from COVID-19 convalescents were analyzed to establish cut-offs for seropositivity. A double cut-off of rMFI >5 for anti-RBD and anti-Spike FL yielded a specificity of 99.7% and a sensitivity of 95% (27). Serum from an individual who had received three doses of the Pfizer/BioNTech anti-COVID-19 vaccine was used as standard to convert signals to binding antibody units per millilitre (BAU/mL).

### 2.5 Flow cytometry

PBMC were first washed in FACS Wash Buffer [PBS1X w/o Ca++ and Mg++ (Gibco # 10010023) supplemented with 1% bovine serum albumin (VWR, #K719)] and stained for 10 minutes in the dark in 150μL of cold PBS containing Fixable Near IR Live/Dead viability stain (Molecular Probes, # L34976) for dead cell exclusion. Cells were then washed once in cold PBS1x and incubated for 30 minutes on ice in 50µL of FACS Wash Buffer containing the following fluorochrome-conjugated antibodies: BV605 anti-human CD3 (Clone SK7; BD Biosciences, # 563219), PerCP-Cy5.5 anti-human CD4 (Clone OKT4; Biolegend, # 317428), and Alexa Fluor 488 anti-human CD8 (Clone OKT8; Invitrogen, # 53-0086-42). After incubation and washing, cells were permeabilized in 150µL BD Cytofix/Cytoperm solution for 30 minutes at room temperature and then washed twice in 200µL of 1x BD perm/wash solution (BD Biosciences Fixation and permeabilization kit; # 554714). Intracellular staining was performed in 50µL of 1x BD perm/wash solution containing the following fluorochrome-conjugated antibodies: APC anti-human CD137 (Clone 4B4-1; BD Biosciences, # 550890), BV711 anti-human CD40L (Clone 24-31; Biolegend, # 310837), PE anti-human IFN-γ (Clone 4S.B3; Biolegend, # 502509), and BV421 anti-human TNFA (Clone MAb11; BD Biosciences, # 562783) according to manufactures instructions. Cells were washed one more time in PBS, resuspended, and acquired on an Attune NxT Flow Cytometer (Thermo Fisher).

### 2.6 T cell activation assay

Quantification of T cell activation was performed by flow cytometry as described (28, 29). Briefly, PBMC were thawed, washed twice, and resuspended in RPMI 1640 medium with GlutaMAX™ supplement (Thermo Fisher Scientific, # 61870-010), 1mmol/L Sodium Pyruvate (Gibco # 11360-039), 1mmol/L MEM NEAA (Gibco # 11140-035), 50nmol/L 1-thioglycerol (Sigma-Aldrich, # M1753), 12μg/mL Gentamycin (VWR, # E737), and 10% heat-inactivated Foetal Bovine Serum (Gibco, # 10270-106). Cells were plated in a 96-well round bottom cell culture plate (1M cells in 200μL per well), stimulated for 3 hours with Spike-I or Spike-C pool solutions (8μl), or NOI pools (1.5μg/mL final concentration per peptide). Cells were then further incubated for 18h after addition of Brefeldin A/Monensin cocktail (GolgiStop 500X, Invitrogen # 00-4980-93). At the end of the incubation, the plate was placed on ice, and cell pellets washed once in cold PBS before flow cytometry processing.

### 2.7 T cell phenotypic assessment

To evaluate T cell reactivity to antigenic challenge, a Reactivity Score (RS) metric was generated from the analysis of cell populations defined by the combined expression of 4 AIMs. These markers and 3 gated areas of interest (Single +, Double +, and “All”) were used to define a total of 16 partially overlapping cell populations (**Supplementary Figure S1A**, **Supplementary Table S2**). We selected T cell populations whose response (frequency after stimulus minus background frequency) was significantly higher in healthy controls than HSCT patients and normalized each value by the average frequency of all measurements for that population. When the frequency after stimulation was smaller than the background (frequency after stimulus minus background frequency < 0), the patient was assigned a RS value of ‘zero’. The RS for each patient was then computed by averaging the normalized frequency of the selected populations for each T cell subset (selected T cell populations are shown in **Supplementary Figure S2**). Similarly, the score for differential reactivity (DR) was computed by normalizing the difference in T cell response to Spike-C and Spike-I pool stimulation (Spike-C minus Spike-I). The T cell populations selected for DR calculation are shown in **Supplementary Table S2**. Raw frequency data for each population are reported in **Supplementary Table S3**.

### 2.8 Statistical analysis

Statistical analyses were performed using GraphPad Prism V.8 (GraphPad software). Two-tailed Fisher’s Exact test, Binomial test, Wilcoxon matched pairs signed rank test, and Mann-Whitney test were used when appropriate. The Spearman’s rank correlation coefficient was computed to assess the correlation between variables.

## 3 Results

### 3.1 Patient cohort

Peripheral blood mononuclear cells (PBMC) and serum samples were collected between March 2021 and February 2022 from 48 HSCT recipients 4 weeks after receiving two doses of Moderna/mRNA-1273 or Pfizer/BioNTech BNT162b mRNA vaccines (see **Supplementary Table S1** for an overview of the patients). The median time from transplantation was 435 d [IQR, 273-975]. Of the 48 HSCT patients, 26 received continued immunosuppression 6 months after transplantation due to GvHD (a high rate, as many patients were recruited from the GvHD clinic). As of January 2023, only one patient developed severe COVID-19 requiring hospitalization with mechanical ventilation after Delta VOC infection.

### 3.2 Seroconversion in HSCT recipients following 2 doses of mRNA vaccines

**Figure 1:**
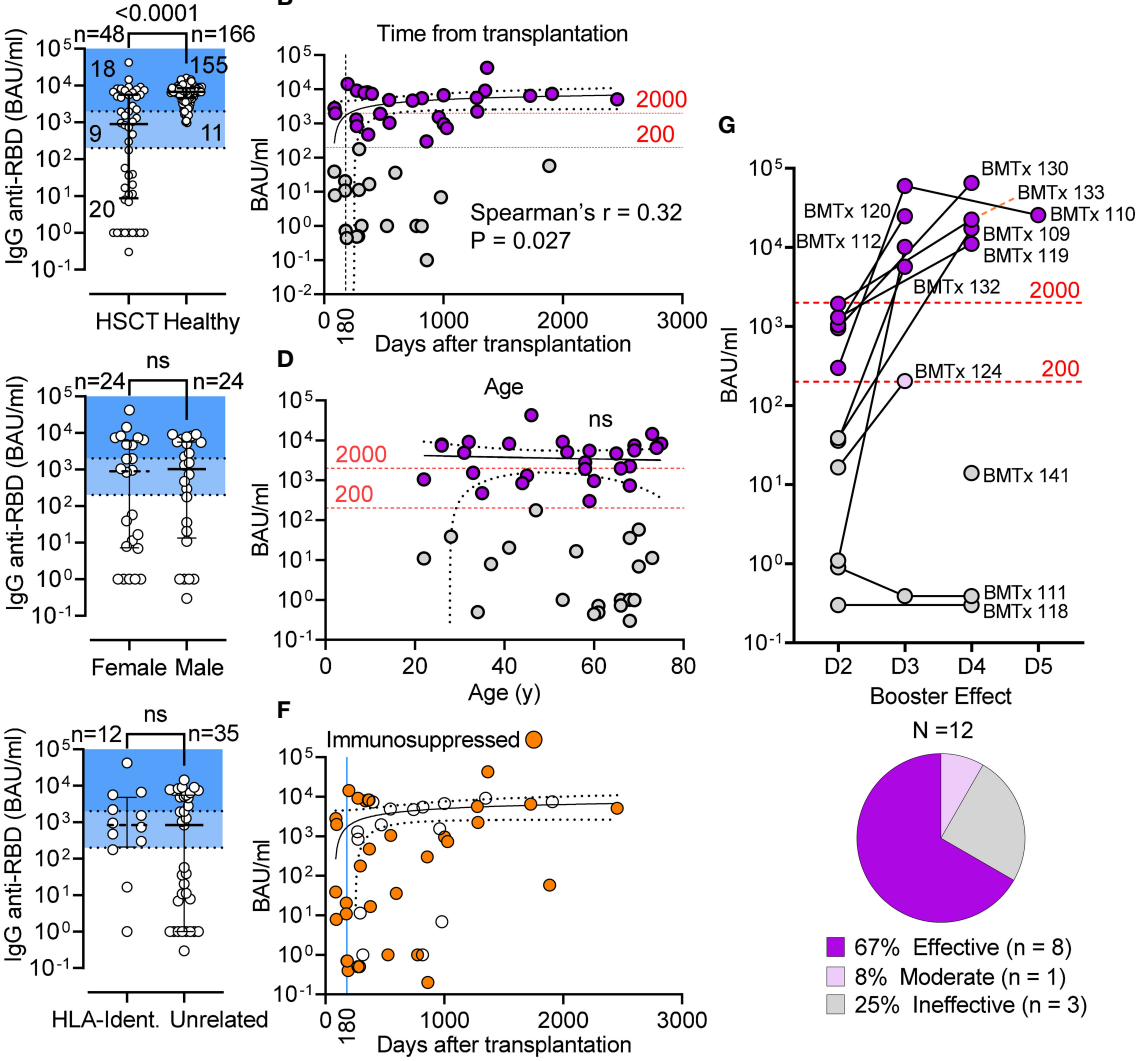
Seroconversion in HSCT patients following 2 doses of mRNA vaccines. **(A)** IgG anti-RBD (BAU/mL) level in HSCT recipients and healthy controls; median and IQR are shown. Shaded areas denote seroconverts (>200 BAU/mL) and strong responders (>2000 BAU/mL). **(B)** Correlative relationship between IgG anti-RBD (BAU/mL) and time from transplantation in 48 HSCT patients (Spearman’s r = 0.32, P = 0.027). Standard immunosuppressive therapy is discontinued 180 d after transplantation. **(C)** IgG anti-RBD (BAU/mL) in female v. male HSCT recipients; median and IQR are shown; Mann-Whitney test (not significant). **(D)** Correlative relationship between antibody level (BAU/mL) and patient age (not significant). **(E)** anti-S-RBD IgG levels in patient receiving BM from related and unrelated donor; Mann-Whitney test (not significant). **(F)** Patients receiving immunosuppressive therapy are shown as in graph B (BAU/mL v days from transplantation). **(G)** anti-S-RBD IgG levels after the 3rd (D3), 4th (D4) and 5th (D5) dose. Pie chart shows breakdown of booster efficacy. The 95% confidence band is shown for panels **(B, D, F)** BAU: binding antibody units. ns, not significant.

Following vaccination, the concentration of serum IgG anti-RBD in HSCT patients was significantly lower than in healthy controls (P < 0.0001; **Figure 1A**) with a median IgG anti-RBD level of 898 BAU/mL [IQR 10-5842] in the HSTC group, and 6677 BAU/mL [IQR 4358-8395] in controls. As shown in **Figure 1A**, only 37.5% (18/48) of patients had protective levels of IgG anti-RBD (>2000 BAU/mL), 21% (10/48) had a clear response (200-2000 BAU/mL), 10% (5/48) had low response (20-200 BAU/mL), while the remainder 31% (15/48) did not seroconvert (0-20 BAU/mL). We found a positive correlation between IgG anti-RBD levels and the time after transplantation (Spearman r = 0.32, P = 0.027; **Figure 1B**), but no correlation between the magnitude of response and gender or age (**Figures 1C, D**). This cohort included patients with high risk of severe COVID-19 who were receiving immunosuppression for GvHD. Nevertheless, we found no significant difference in response between recipients of cells from HLA-identical siblings and those from unrelated donors (**Figure 1E**), and no impact of immunosuppressive therapy on vaccine response (Fisher’s Exact, P = 0.236; **Figure 1F** and data not shown). Notwithstanding the magnitude of response after the second dose, 8 of 12 double-vaccinated patients (67%) for whom longitudinal serology data were available benefited from a 3^rd^ and a 4^th^ dose, 1 patient showed suboptimal response after the 3^rd^ dose, and 3 non-responders (25%) failed to seroconvert even after the 3^rd^ or 4^th^ dose (**Figure 1G**). In this sub-cohort, the median time for serum IgG assessment after vaccination was 4.5, 8, and 5 weeks for the 2^nd^, 3^rd^, and 4^th^ dose, respectively. Of note, 3 of these 8 patients showed positive serological results for the IgG anti-nucleocapsid (not shown), indicating that the higher IgG anti-RBD levels measured were the likely result of hybrid immunity. These non-responders had developed severe GvHD or relapse of malignancy.

### 3.3 T cell response to immunodominant SARS-CoV-2 Spike peptides

**Figure 2:**
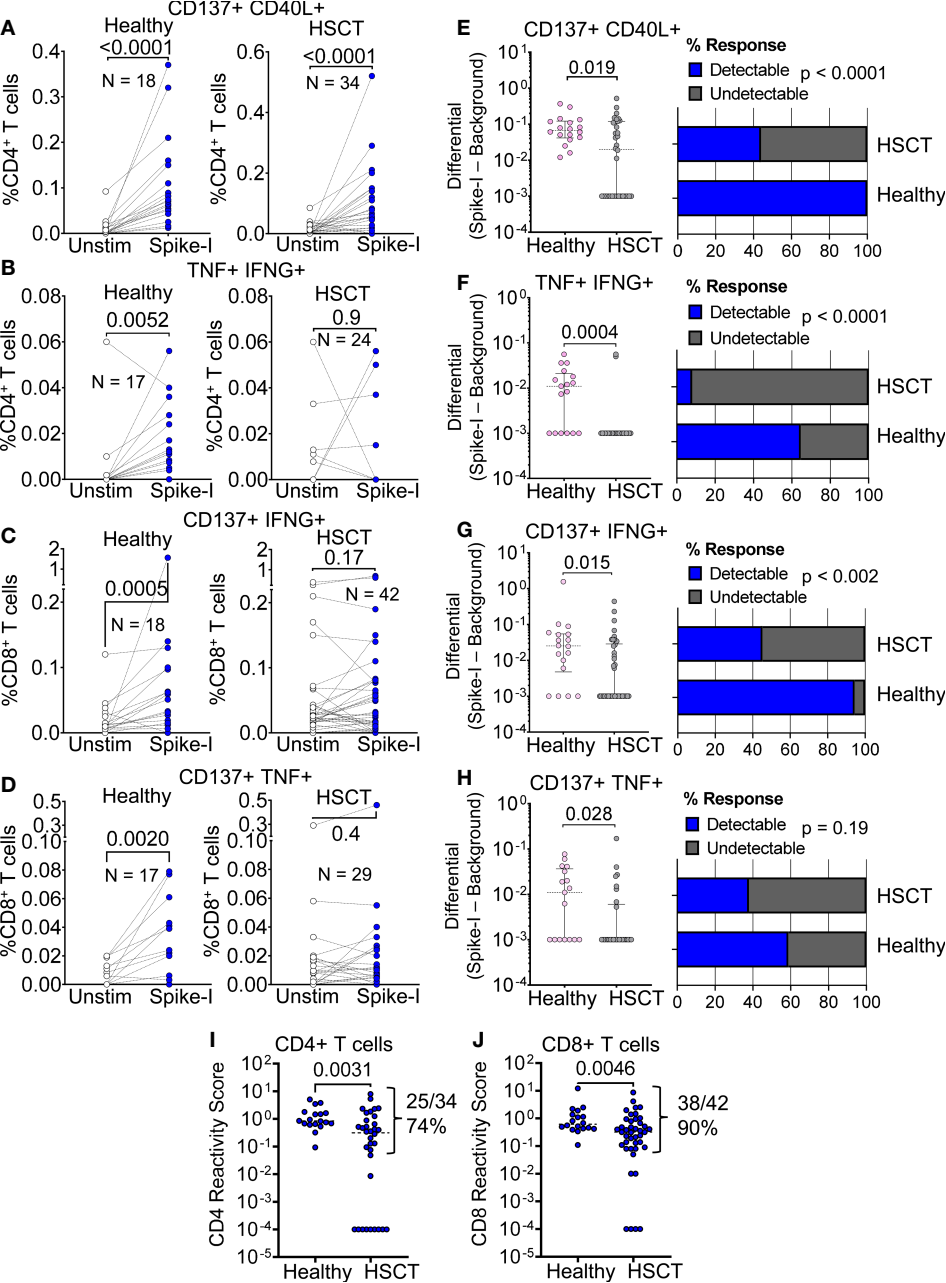
T cell response to immunodominant SARS-CoV-2 Spike peptides. **(A–D)** Flow cytometric quantification of **(A)** CD137^+^ CD40L^+^ CD4^+^ **(B)** TNF^+^ IFN-γ^+^ CD4^+^, **(C)** CD137^+^ IFN-γ^+^ CD8^+^, and **(D)** CD137^+^ TNF^+^ CD8^+^ T cell populations in healthy and HSCT patients after stimulation with immunodominant S protein epitopes (Spike-I pool). Unstim. = background level of the T cell population; Wilcoxon matched pairs signed rank test; P values are shown. **(E–H)** Magnitude of response after stimulation with the Spike-I pool (Spike-I minus Background) in healthy controls vs. HSCT patients for the same populations shown in **(A–D)**; Mann-Whitney test; P values are shown. The percentage of subjects with detectable response is shown on the right (blue columns); Binomial test, P values are shown. **(I, J)** Comparison of T cell Reactivity Scores between healthy subjects and HSCT patients for **(I)** CD4^+^ T cells and **(J)** CD8^+^ T cells, Mann-Whitney test; P values are shown.

We first examined spike peptide-specific T cell responses towards a pool of immunodominant SARS-CoV-2 peptides (Spike-I pool, see Methods). To measure the response, we analyzed the activation of CD4^+^ Th cells and CD8^+^ CTL using activation-induced markers (AIMs) gated as illustrated in **Supplementary Figure S1A** and Methods. We found that the CD4^+^/CD8^+^ T cell ratio was significantly lower than in healthy donors (0.265 vs. 0.894 median values; P < 0.0001; **Supplementary Figure S1B**), a common observation in patients that have undergone bone marrow transplantation. Of 34 HSCT patients tested for T cell reactivity, 56% (19/34) had increased Spike-I specific CD4^+^ Th cells, as measured by CD137 and CD40L markers (**Figure 2A**), a response significantly lower than in controls where 18/18 responded (P = 0.019; **Figure 2E**). Similarly, we found a lower frequency of Spike-I specific TNF^+^ IFN-γ^+^ CD4^+^ Th cells (**Figure 2B**) with responses detected in only 8.3% of patients analyzed with this marker combination (P = 0.0004; **Figure 2F**) as well as a lower response of Spike-I specific CD8^+^ T cells expressing CD137 and IFN-γ (**Figure 2C**), or CD137 and TNF (**Figure 2D**), which were observed in 43% (18/42) (P = 0.015; **Figure 2G**), and 28% (8/29) (P = 0.028; **Figure 2H**) of patients, respectively. Because we found some individual variation in the response patterns of the CD137, CD40L, TNF, and IFN-γ markers in both CD4^+^ and CD8^+^ T cell subsets, we used the reactivity score (RS) metric, a normalized value that incorporates the contribution of all relevant combinations of these 4 AIMs to provide a more unbiased quantification of reactivity (see methods and **Supplementary Figure S1A**) and confirmed that overall Spike-I specific response was significantly reduced in both the CD4^+^ and CD8^+^ T cell compartment of HSCT patients (see patients’ ranking by RS in **Supplementary Figures S2A, B**). Accordingly, comparison of RS values confirmed lower reactivity in HSCT patients for both the CD4^+^ (median 0.842 vs 0.315, P = 0.0031; **Figure 2I**) and CD8^+^ T cell subset (median 0.62 vs 0.32 value; P = 0.0046; **Figure 2J**). Nonetheless, CD4^+^ and CD8^+^ T cell response to the epitopes from the immunodominant regions of the S protein remained undetectable in 26% and 10% of HSCT patients, respectively.

### 3.4 T cell response to peptides spanning the entire SARS-CoV-2 Spike protein

**Figure 3:**
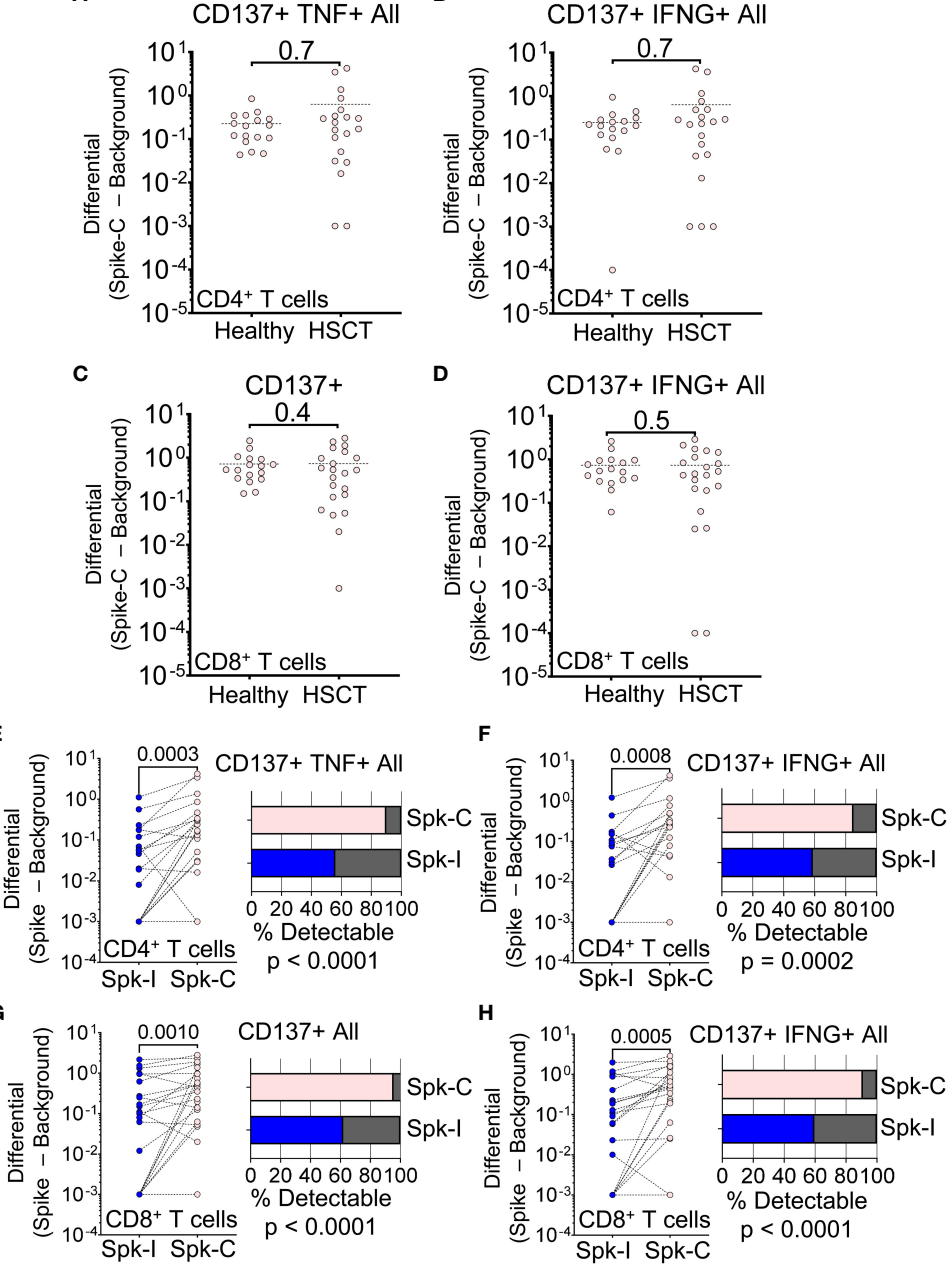
T cell response to peptides spanning the entire SARS-CoV-2 spike protein. **(A–D)** Magnitude of response after stimulation with the Spike-C pool (Spike-C minus Background) for **(A)** CD137+ TNF+ CD4+ All, **(B)** CD137+ IFN-g+ CD4+ All, **(C)** CD137+ CD8+, and **(D)** CD137+ IFN-g+ CD8+ All T cell populations in healthy vs. HSCT patients; Mann-Whitney test (not significant). **(E–H)** Difference in Spike-I vs. Spike-C response for the same T cell populations shown in panels **(A–D)**; Mann-Whitney test, P values are shown. The fraction of patients with detectable response is shown on the right; Binomial test; P values are shown. Spike-I: immunodominant S protein epitopes; Spike-C: epitopes spanning the entire S protein.

To ensure that we did not overlook any T cell specificities, we also tested overlapping peptides that covered the complete spike protein (Spike-C pool). Surprisingly, the response of HSCT recipients to wider antigenic stimulation was much improved compared to the immunodominant spike peptide pool. In fact, HSCT recipients and controls were not significantly different in terms of changes in frequency of CD137^+^ TNF^+^ and CD137^+^ IFN-γ^+^ CD4^+^ T cell (P = 0.7; **Figures 3A, B**) or CD137^+^ and IFN-γ^+^ CD8^+^ T cell populations (P > 0.4; **Figures 3C, D**). We observed better response to Spike-C stimulation for CD137^+^ TNF^+^ (P = 0.0003, Wilcoxon ranked test; **Figure 3E**) and CD137^+^ IFN-γ^+^ (P = 0.0008; **Figure 3F**) CD4^+^ cells, and an increase in the fraction of patients with detectable response (90% and 85% for the two cell populations, respectively; P < 0.0001 and P = 0.0002, Binomial test; **Figures 3E, F**). CD8^+^ T cells showed similar trends, with better response to Spike-C for cells expressing CD137^+^ alone (P = 0.001; **Figure 3G**) and in combination with IFN-γ^+^ (P = 0.0005; **Figure 3H**), and a response rate of 95% and 91%, respectively (P < 0.0001, Binomial test; **Figures 3G, H**). Altogether, these results suggest that after vaccination HSCT recipients developed cell-mediated immunity with different T cell specificities.

### 3.5 Difference in T cell specificities between HSCT patients and healthy controls

**Figure 4:**
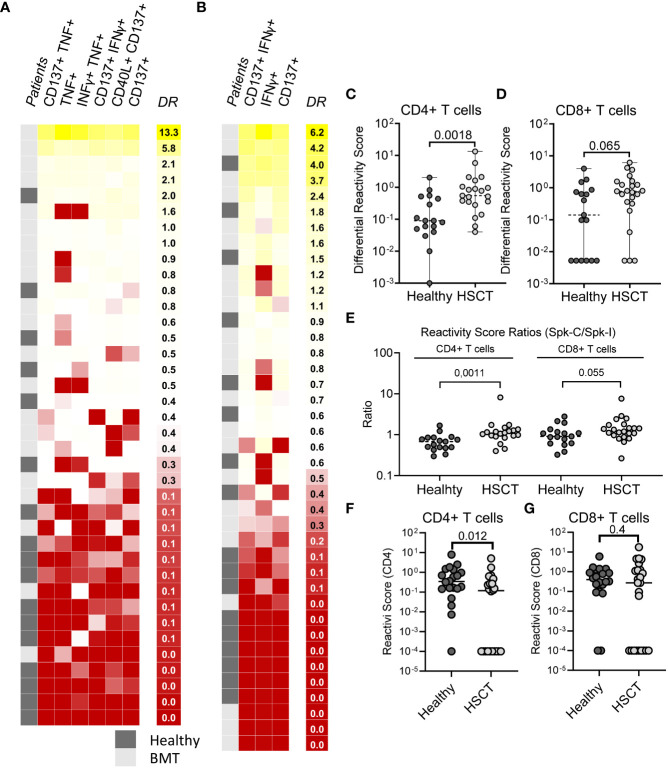
Difference in T cell specificities between HSCT patients and healthy controls. **(A)** Differential Reactivity heatmap of CD4^+^ T cells in healthy (N = 18) and HSCT (N = 21) patients. **(B)** Differential Reactivity heatmap of CD8^+^ T cells in healthy (N = 17) and HSCT (N = 23) patients. Subjects are ranked according to their Differential Reactivity score (highest to lowest). **(C, D)** Differential Reactivity scores of healthy subjects and HSCT patients for **(C)** CD4^+^ and **(D)** CD8^+^ T cells. **(E)** Comparison of Reactivity Score ratios (Spike-C RS/Spike-I RS) between healthy subjects and HSCT patients for CD4^+^ and CD8^+^ T cells. **(F, G)** Reactivity Scores for **(F)** CD4^+^ and **(G)** CD8^+^ T cells in healthy vs. HSCT patients following stimulation with NOI’s random pools; Mann-Whitney test; P values are shown.

To compare differences in stimulus-specific T cell response (T cell specificity) between cohorts we defined a score that reflects differential reactivity (DR) to Spike-I and Spike-C stimulation (see Methods for details). A greater fraction of HSCT recipients showed DR values higher than controls for both CD4^+^ (**Figure 4A**) and CD8^+^ (**Figure 4B**) subsets indicating that the response to Spike-C was generally stronger in patients than controls. The HSCT cohort had higher DR for CD4^+^ T cells (median DR = 0.55 vs. 0.090; P = 0.018; **Figure 4C**), but not for CD8^+^ T cells (median DR = 0.78 vs. 0.14; P = 0.065; **Figure 4D**). Likewise, the ratio of the RS of Spike-C and Spike-I (Spike-C RS/Spike-I RS) was significantly higher in the HSCT cohort than controls for CD4^+^ T cells (median ratio of 1.1 vs. 0.68; P = 0.0011; **Figure 4E**, left), but not CD8^+^ T cells (1.3 vs 0.91, P = 0.055; **Figure 4E**, right), indicating a difference in specificities between the two T cell subsets. This distinction could be explained in part by the fact that the pools are composed of 15-mers peptides which preferentially activate CD4^+^ T cells. For a closer analysis of CD8^+^ T cells, we tested response to the NEC OncoImmunity (NOI) pool, a collection of 9-10-mers S protein epitopes optimized for the detection of spike-specific CD8^+^ T cells - see Methods and (25). HSCT patients had reduced CD4^+^ T cell reactivity after stimulation with the NOI pool (0.34 vs. 0.12; P = 0.012; **Figure 4F**), but the response of the CD8^+^ T cell subset was retained (0.40 vs. 0.27; P = 0.4; **Figure 4G**). Altogether, these results suggest that the skewed peptide specificity profile within the CD4^+^ T cell compartment is unique to HSCT recipients.

### 3.6 Relationship between T cell reactivity and antibody level in HSCT patients

**Figure 5:**
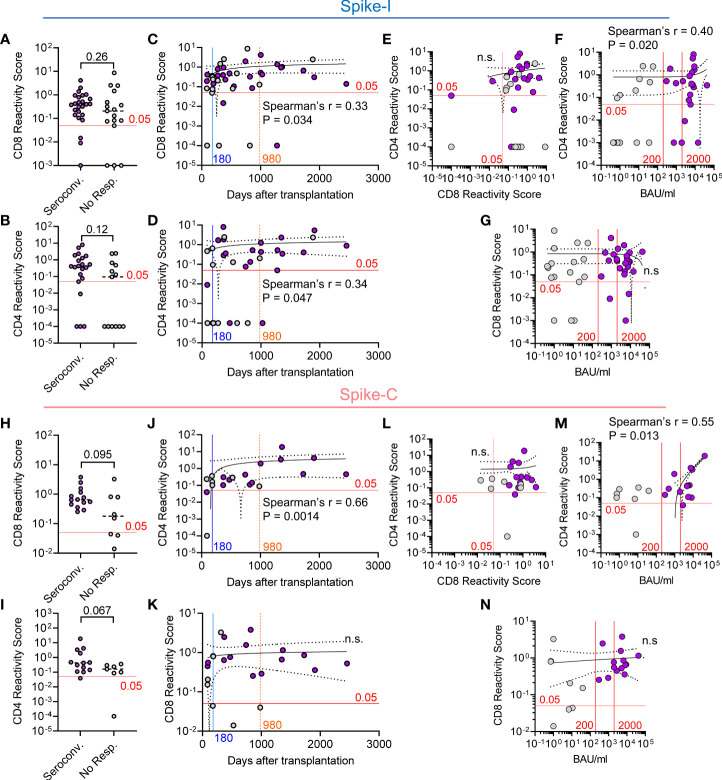
Relationship between T cell reactivity and antibody level in HSCT patients. **(A, B)** Comparison of the Reactivity Scores between seroconverted and non-responders for **(A)** CD8^+^ and **(B)** CD4^+^ T cells following stimulation with Spike-I peptides. Mann-Whitney test, P values are shown (not significant). **(C, D)** Correlative relationship between time from transplantation and the Reactivity Scores of **(C)** CD8^+^ and **(D)** CD4^+^ T cells following stimulation with Spike-I peptides. **(E)** Correlation between CD4 and CD8 Reactivity Scores. **(F, G)** Correlation between anti-S-RBD IgG level (BAU/mL) and **(F)** CD4 or **(G)** CD8 Reactivity Scores. **(H, I)** Comparison of Reactivity Scores between seroconverted and non-responders for **(H)** CD8^+^ and **(I)** CD4^+^ T cells following stimulation with Spike-C peptides. Mann-Whitney test, P values are shown. **(J, K)** Correlative relationship between time from transplantation and the Reactivity Scores of **(J)** CD4^+^ or **(K)** CD8^+^ T cells following stimulation with Spike-C peptides. **(L)** Correlation between CD4 and CD8 Reactivity Scores in HSCT patients. **(M, N)** Correlation between anti-S-RBD IgG level and **(M)** CD4 or **(N)** CD8 Reactivity Scores. The red line at 0.05 RS units represents the empirical threshold for positive response. Spearman’s coefficient r, P values, and 95% confidence bands are shown. ns, not significant, Purple dots denote seroconverted patients.

Next, we investigated the relationship between humoral response and T cell immunity in the context of both Spike-I and Spike-C stimulation. Seroconverted and non-responders had similar reactivity to Spike-I for both CD8^+^ (median RS of 0.40 vs. 0.21; P = 0.26; **Figure 5A**) and the CD4^+^ T cells (median RS of 0.40 vs. 0.094; P = 0.12; **Figure 5B**). Moreover, time from transplantation was predictive of T cell reactivity for CD8^+^ (Spearman’s r = 0.33, P = 0.034; **Figure 5C**) and CD4^+^ T cells (Spearman’s r = 0.34, P = 0.047; **Figure 5D**), even though there was no association between the responses of the two T cell subsets (Spearman’s r = 0.28, P = 0.11, ns; **Figure 5E**). Moreover, IgG anti-RBD level correlated with the reactivity of CD4^+^ (Spearman’s r = 0.40; P = 0.020; **Figure 5F**), but not CD8^+^ T cells (**Figure 5G**). Likewise, T cell reactivity in seroconverted and non-responders following Spike-C stimulation were not significantly different in both the CD8^+^ (median RS of 0.65 vs. 0.18; P = 0.095; **Figure 5H**), or the CD4^+^ T cell subset (median RS of 0.43 vs. 0.16; P = 0.067; **Figure 5I**). However, Spike-C stimulation revealed a strong correlation between the time after transplantation and CD4^+^ T cell reactivity (Spearman’s r = 0.66; P = 0.0014; **Figure 5J**), which was not observed for the CD8^+^ T cell compartment (**Figure 5K**). We found no correlation between CD4^+^ and CD8^+^ T cell reactivity to Spike-C (**Figure 5L**), while IgG anti-RBD level correlated with the reactivity of CD4^+^ T cells (Spearman’s r = 0.55; P = 0.013; **Figure 5M**), but not CD8^+^ T cells (**Figure 5N**). Altogether, these data suggest that T cell response precedes humoral immunity and that the response to vaccination of CD4^+^ but not CD8^+^ T cells is associated to antibody level and improves with time after transplantation.

## 4 Discussion

Prior evidence has suggested that HSCT patients have poor rates of seroconversion after vaccination and an associated risk of severe COVID-19 disease and death (22, 23, 30–32). While the seroconversion rate in our cohort was also poor, as only 37.5% of HSCT recipients developed the protective IgG anti-RBD levels typically seen in most vaccinated controls (>2000 BAU/mL), only 3 HSCT recipients did not seroconvert after a 3^rd^ and 4^th^ dose. In contrast, most patients responded with spike-specific T cell immunity, which was observed in 90% of the patients for both CD8^+^ and CD4^+^ T cells. Although a limited number of patients were examined in this study, the inclusion of high-risk subjects with GvHD on immunosuppressive therapy that could influence response to the vaccine and the observation that only one patient was hospitalized for severe COVID-19 support the notion that vaccine-induced T cell immunity protected these HSCT patients against severe disease.

As a key component of the immune response against viral infections (33), T cell-mediated immunity plays a major role in the control of MERS and SARS-CoV-1 infections (34, 35) as well as in the protection of patients receiving B cell-depleting therapies (36). Although T cell immunity is crucial for protection from COVID-19 disease (37–39) and it has been observed in asymptomatic and mild SARS-CoV-2 infections (40), its involvement in the response to vaccination of HSCT patients remains unclear. One study reported a weak vaccine-induced T cell immunity which was linked to an insufficient humoral responses (23), and another showed limited T cell response in patients at less than 2 years after HSCT (22). These results are in contrast with studies showing that most humoral vaccine responders displayed CD4^+^ T cell immunity (21, 41) as well as an early, small study reporting that 52% and 70% of vaccinated HSCT recipients showed CD8^+^ and CD4^+^ T cell responses, respectively (20). However, the patients in the latter study had a median time of 55 months after transplantation and the frequency of spike-specific responders was low. An additional study reported lower response frequency in comparison to chronic myeloid leukaemia patients (42) and another detected T cell responses (> 30 IFN-γ releasing cells/10^5^ cells), but failed to dissect the relative contribution of the CD4^+^ and CD8^+^ T cell subsets (24). The impact of vaccine T cell immunity in breakthrough infections in transplanted patients has still not been demonstrated, but it has been recently suggested that T cell immune responses correlate with protection in organ transplanted patients, to compensate for the suboptimal antibody response (43).

To quantify the extent of CD4^+^ and CD8^+^ T cell involvement in a more comprehensive and unbiased way, we generated a reactivity score (RS) metric that incorporates the contribution of all relevant populations defined by 4 AIMs commonly used to assess T cell reactivity (21, 29, 40, 44, 45). The data revealed that the reactivity of T cells to the immunodominant regions of the S protein was lower in HSCT patients than in controls for both cell types. In contrast, superior T cell responses were detected after challenge with a complete spike peptide pool, demonstrating a skewed T cell response profile, not favouring the immunodominant peptide sets. Importantly, skewed reactivity was particularly significant for the CD4^+^ T cell subset, which may not only reflect intrinsic differences between the CD4^+^ and CD8^+^ T cell response to COVID-19 (46) but also be a direct consequence of the slow functional recovery of engrafted T helper cells, a process that may require years to complete (47, 48). It will be interesting to test the usefulness of the RS metric in the assessment of SARS-CoV-2 protection in other vaccinated cohorts, especially in comparison or conjunction with other metrics such as CD4 counts (49). Moreover, our cohort was characterized by a significant reduction in the CD4^+^/CD8^+^ T cell ratio supporting the observation that the dynamics of T cell antigenic repertoire reconstitution in HSCT recipients are complex (47, 48). One hypothesis to explain the preferential reactivity of HSCT recipients to less-immunogenic regions of the S protein is that pre-existing cross reactivity to seasonal coronaviruses (50) can be adoptively transferred to the host and thereafter reshaped during the immune reconstitution process. This notion must however be based on two key assumptions: 1) the immunodominant epitopes should preferentially cross-react and stimulate T cell clones selected from previous exposures to seasonal coronaviruses (51) and 2) selection pressures taking place during immune reconstitution must favour clones with higher specificity to non-immunogenic regions. In this regard, it is also important to note that some patients may have received HSCT from vaccinated donors, which may further impact clonal selection dynamics during immune reconstitution. Alternatively, vaccines may have preferentially expanded seasonal coronaviruses cross-reactive host T cells. Further experimental work will be necessary to elucidate the molecular and evolutive processes at the origin of this phenomenon (52).

Notwithstanding the type of stimulation, it is important to note that T cell response was detected independently of seroconversion even though T helper cell reactivity was fully restored in correlation with the humoral response in patients, including HSCT recipients transplanted up to 2455 days prior to the assessment of vaccine immunity. On the contrary, the degree of CD8^+^ T cell response was not affected by the time of transplantation or antibody level, demonstrating vaccine responses regardless of serological response. This is in line with the fact that the cytotoxic T cell response develops rapidly after infection and it is associated to mild symptomatology and virus clearance (53, 54). Because the pools used in these tests were composed of 15-mers peptides, which preferentially activate CD4^+^ T cells, we further tested response using 9-10-mers peptides with greater specificity for CD8^+^ T cells and confirmed that virus-specific cytotoxic T cell immunity is retained in these patients. Overall, the response pattern in terms of coordination between cell-mediated and antibody-mediated immunity was heterogeneous (21), similarly to what has been reported for SARS-CoV-2 infections (55).

Overall, current data indicate that cell-mediated immunity plays a crucial protective role in patients with certain immunodeficiencies or individuals under specific immunosuppressive therapies. It is worth noting that T cell response does not necessarily correlate with serological measures of protection. Multiple studies have demonstrated compromised vaccine-induced immunization and long-term efficacy of SARS-CoV-2 vaccines in immunosuppressed patients. This includes individuals with rheumatic disease, solid organ transplant recipients (including kidney transplant recipients on dialysis), and cancer patients with haematological malignancies or solid tumours (56–58). However, the impact of immunodeficiency conditions and specific immunosuppressive therapy and the interaction between T cell and humoral immune responses may vary. For instance, solid-organ transplant recipients are at a particularly higher risk of severe COVID-19 infections and mortality. They exhibited significant defects in both vaccine-induced humoral (59) and T cell-mediated immune response (21). Cancer patients with haematological malignancies or solid tumours also experience concurrent suppression of both arms of the adaptive immune system. In addition to impaired vaccine-induced seroconversion (60, 61), these patients often displayed reduced SARS-CoV-2-induced CD4^+^ and CD8^+^ T cell responses (36, 62), and impaired T cell responses associated to the use of checkpoint inhibitors (CPIs) (63). This level of coordinated immunosuppression was not observed in other conditions, such as X-linked agammaglobulinemia (XLA), where patients who lack mature B cells could still retain functional spike-specific cell-mediate immunity (21). Moreover, we and others have observed good vaccine-specific T cell responses in rituximab-treated patients with rheumatoid arthritis (27) or multiple sclerosis (64, 65) even in absence of seroconversion. This demonstrates that cellular immunity can develop even without detectable serological response. Interestingly, we have also observed that T cell and serological response can be compromised in MS patients taking fingolimod, another disease-modifying agent, indicating that the inhibitory mechanism of the immunosuppressive therapy impacts the characteristics of the immunological response to the vaccine (65).

Differences in the degree of response between T cell subsets observed in our study have also been reported in other cohorts of immunocompromised individuals. For example, a study on people living with HIV (PLWH) with uncontrolled viremia, a category of patients at elevated risk of severe COVID-19-related infection and death, reported specific CD4^+^ T cell immunity following vaccination, but impaired CD8^+^ T cell function (66). Similarly, evidence from other high-risk immunocompromised patient groups, such as diabetic individuals with poor glycaemic control, shows a normal response after the second dose of vaccine in terms of neutralizing antibody production, as well as CD4^+^ and CD8^+^ T cell responses (67, 68).

In summary, we have shown that while vaccine-induced CD4^+^ T cell response in HCST recipients improves over time and it is associated to antibody response, CD8^+^ T cell function is minimally affected by the immune reconstitution posttransplant and contributes to protection from severe COVID-19 disease. The skewed peptide reactivity profile in the HSCT recipients remains unexplained but may relate to the clonal composition of vaccine-expanded T cells.

## Data availability statement

The original contributions presented in the study are included in the article/**Supplementary Material**. Further inquiries can be directed to the corresponding authors.

## Ethics statement

The studies involving human participants were reviewed and approved by Health Region South-East Regional Ethics committee. The patients/participants provided their written informed consent to participate in this study.

## Author contributions

LF, TAT, TG-D, and LM designed the study. LF, MG, JO, VC, KL, LT, and TT performed experiments and preliminary analyses of data. FL-J designed the antibody analyses and analyzed serological data. All authors contributed to data analyses. TAT, AM, TG-D handled patients and clinical information. RS, ST, BM, TC performed predictions of immunodominant Class I-restricted peptides. LF and AM prepared the first draft of the manuscript. All authors contributed to the article and approved the submitted version.
