# Supplementary material for: Robust spike-specific CD4+ and CD8+ T cell responses in SARS-CoV-2 vaccinated hematopoietic cell transplantation recipients: a prospective, cohort study
Source: Front Immunol. 2023 Jul 7;14:1210899. doi: 10.3389/fimmu.2023.1210899 (PMC10369799; doi:10.3389/fimmu.2023.1210899)
Supplement: Supplementary file 2 [file Presentation_1.pdf]

Supplementary Material for:

Robust Spike-specific CD4^{+} and CD8^{+} T cell responses in SARS-CoV-2 vaccinated hematopoietic cell transplantation recipients: a prospective, cohort study

Lorenzo Federico[1,2*], Tor Henrik Anderson Tvedt[3], Murat Gainullin[1,2], Julie Røkke Osen[1,2], Viktoriia Chaban[1,2], Katrine Persgård Lund[1,2], Lisa Tietze[1,4], Trung Tran[1,4], Fridtjof Lund-Johansen[1,4], Hassen Kared[1,2], Andreas Lind[5], John Torgils Vaage[4,6], Richard Stratford[7], Simen Tennøe[7], Brandon Malone[7], Trevor Clancy[7], Anders Eivind Leren Myhre[3,4], Tobias Gedde-Dahl[3*], and Ludvig Andre Munthe[1,2*]

[1] Department of Immunology, Oslo University Hospital, Oslo, Norway, [2] KG Jebsen Centre for B cell Malignancies, Institute of Clinical Medicine, University of Oslo, Norway, [3]Department of Haematology, Oslo University Hospital, Oslo, Norway, [4] ImmunoLingo Convergence Center, Institute of Clinical Medicine, University of Oslo, Oslo, Norway, [5]Department of Microbiology, Oslo University Hospital, Oslo, Norway, [6] Institute of Clinical Medicine, University of Oslo, Oslo, Norway, [7] NEC OncoImmunity AS, Oslo, Norway.

***Corresponding authors:**

Lorenzo Federico (lorenzo.federico@medisin.uio.no), Tobias Gedde-Dahl (tgeddeda@ous-hf.no), Ludvig Andre Munthe (l.a.munthe@medisin.uio.no)

Supplementary Material Summary:

1) **Supplementary Figures.**
    - **Supplementary Figure S1.**
    - **Supplementary Figure S2**
2) **Supplementary Tables.**
    - **Supplementary Table S1.**
    - **Supplementary Table S2**

1. Supplementary Figures.

Supplementary Figure S1. Flow cytometry gating and data acquisition strategy. (A) An example of cell gating of $CD4^+$ and $CD8^+$ T cell populations for healthy and HSCT patients. A schematic representation of the six combinations of the four markers and the three population types (grey quadrants) used in the study is shown (see list of populations in Supplementary Table S2 and analysis details in Methods). (B) Comparison of $CD4^+/CD8^+$ T cell ratio distributions between HSCT (N = 37) and healthy (N = 18) patients ($P < 0.0001$; Mann-Whitney test).

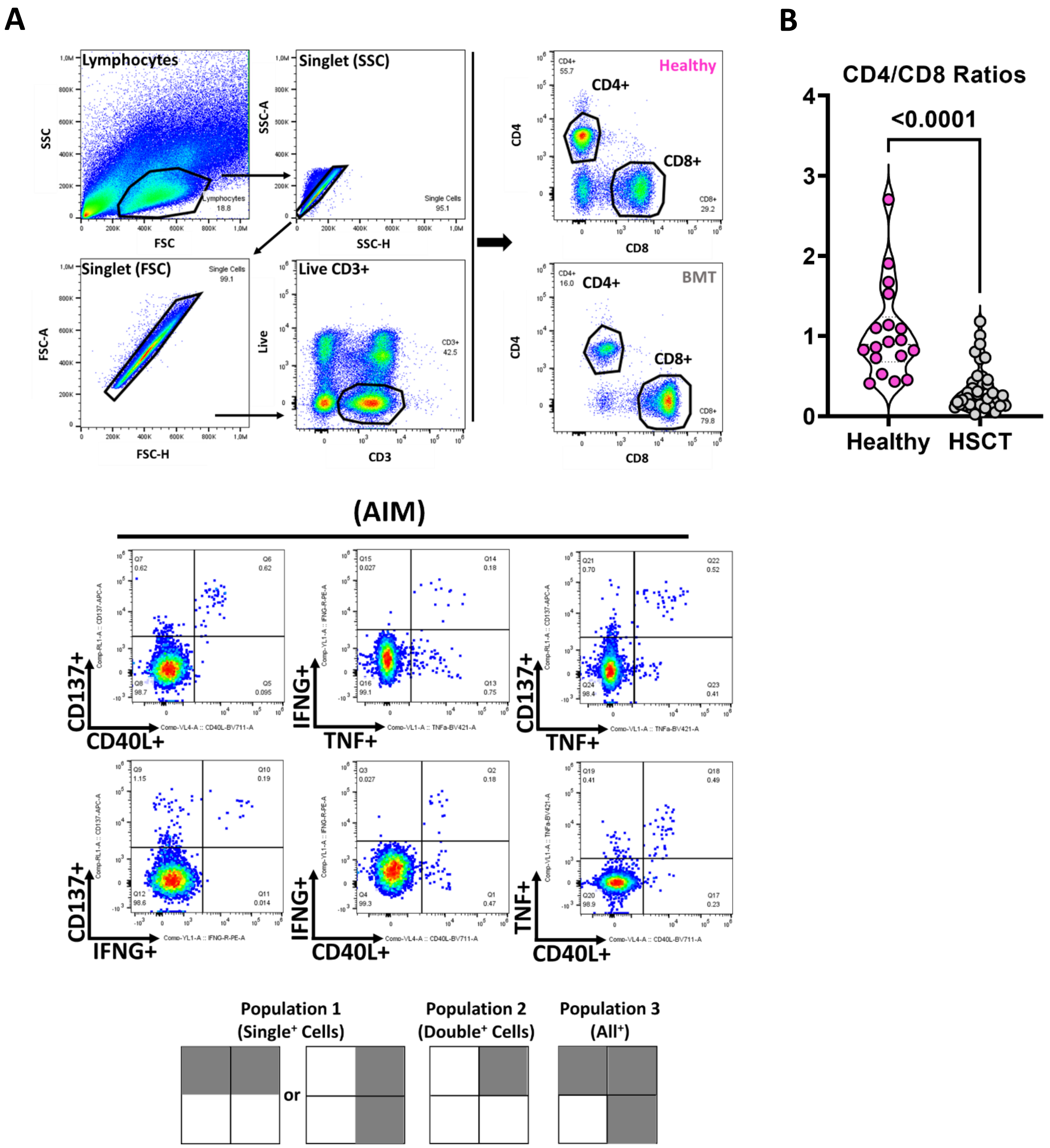
A
B
Lymphocytes
Singlet (SSC)
Singlet (FSC)
Live CD3+
SSC
FSC
SSC-A
SSC-H
FSC-A
FSC-H
Live
CD3
Lymphocytes 18.8
Single Cells 95.1
Single Cells 99.1
CD3+ 42.5
Healthy
BMT
CD4+
CD8+
CD4
CD8
CD4+ 55.7
CD8+ 29.2
CD4+ 16.0
CD8+ 79.8
CD4/CD8 Ratios
<0.0001
Healthy
HSCT
(AIM)
CD137+
CD40L+
IFNG+
TNF+
Population 1
(Single+ Cells)
or
Population 2
(Double+ Cells)
Population 3
(All+)

Supplementary Figure S2. T cell activation heatmap of patient's ranked by reactivity score. (A) Heatmap of $CD4^+$ T cell reactivity after Spike-I stimulation in healthy (N = 18) and HSCT (N = 34) patients. (B) Heatmap of $CD8^+$ T cell reactivity after Spike-I stimulation in healthy (N = 18) and HSCT (N = 42) patients. HSCT recipients and healthy donors are ranked from the highest to the lowest responder according to their Reactivity Score. Striped/greyed out cells represent missing values.

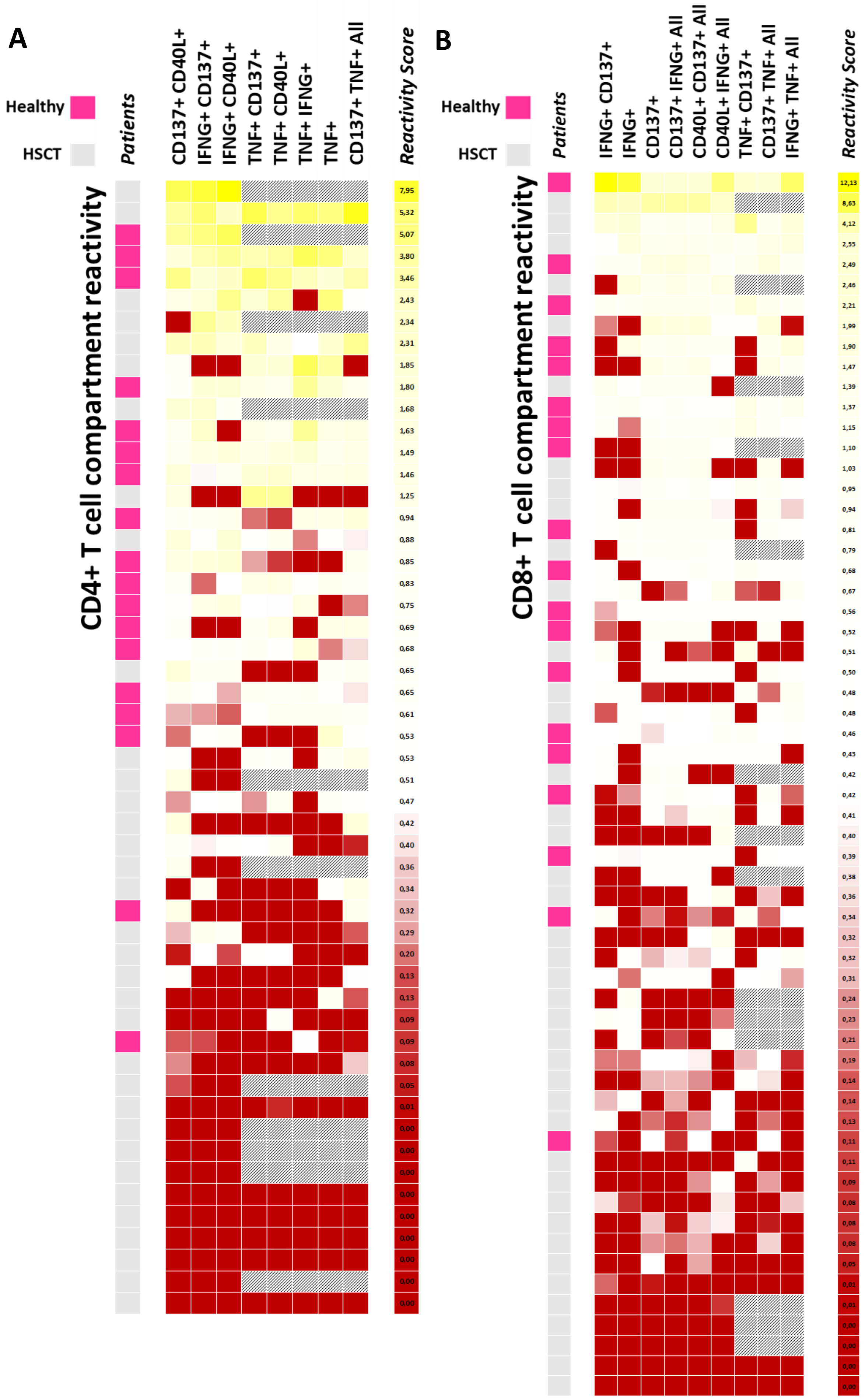
A
Healthy
HSCT
Patients
CD4+ T cell compartment reactivity
CD137+ CD40L+
IFNG+ CD137+
IFNG+ CD40L+
TNF+ CD137+
TNF+ CD40L+
TNF+ IFNG+
TNF+
CD137+ TNF+ All
Reactivity Score
7,95
5,32
5,07
3,80
3,46
2,43
2,34
2,31
1,85
1,80
1,68
1,63
1,49
1,46
1,25
0,94
0,88
0,85
0,83
0,75
0,69
0,68
0,65
0,65
0,61
0,53
0,53
0,51
0,47
0,42
0,40
0,36
0,34
0,32
0,29
0,20
0,13
0,13
0,09
0,09
0,08
0,05
0,01
0,00
0,00
0,00
0,00
0,00
0,00
0,00
0,00
0,00
B
Healthy
HSCT
Patients
CD8+ T cell compartment reactivity
IFNG+ CD137+
IFNG+
CD137+
CD137+ IFNG+ All
CD40L+ CD137+ All
CD40L+ IFNG+ All
TNF+ CD137+
CD137+ TNF+ All
IFNG+ TNF+ All
Reactivity Score
12,13
8,63
4,12
2,55
2,49
2,46
2,21
1,99
1,90
1,47
1,39
1,37
1,15
1,10
1,03
0,95
0,94
0,81
0,79
0,68
0,67
0,56
0,52
0,51
0,50
0,48
0,48
0,46
0,43
0,42
0,42
0,41
0,40
0,39
0,38
0,36
0,34
0,32
0,32
0,31
0,24
0,23
0,21
0,19
0,14
0,14
0,13
0,11
0,11
0,09
0,08
0,08
0,08
0,05
0,01
0,01
0,00
0,00
0,00
0,00

2. **Supplementary Tables.**

Supplementary Table S1. Clinicopathological characteristics of the HSCT cohort.

HSCT cohort (N = 48)

**Age**	*Years*
Median	49
Range	21-73
**Time from transplantation**	*Months*
Median	14
Range	3-82
**Sex**	*n*
Male	24
Female	24
**Diagnose**	*n*
AML	18
MDS	13
ALL	2
Aplastic Anemia	2
Other	13
**Donor Type**	*n*
MUD	35
HLA-identical relative	12
Other	1
**aGVHD**	*n*
Yes	12
No	33
Mild	3
**cGVHD**	*n*
Yes	23
No	25

Abbreviations: MUD, matched-unrelated donor; aGVHD, acute Graft-Versus-Host Disease; cGVHD, chronic Graft-Versus-Host Disease

Supplementary Table S2. CD4$^+$ and CD8$^+$ T cell subpopulations analyzed in the study.

T cell subpopulations (CD4+ and CD8+ T cells)

Marker	T cell Population name		
IFNG	IFNG+	*	**Cell populations single positive for the indicated markers.**
TNF	TNF+	**	
CD137	CD137+	***	
CD40L	CD40L+		
IFNG and TNF	IFNG+ TNF+		**Cell populations double positive for the indicated markers.**
IFNG and CD137	IFNG+ CD137+		
IFNG and CD40L	IFNG+ CD40L+		
TNF and CD137	TNF+ CD137+		
TNF and CD40L	TNF+ CD40L+		
CD137 and CD40L	CD137+ CD40L+		
IFNG and TNF	IFNG+ TNF+ All	**	**Cell populations resulting from the sum of all the populations defined by the indicated markers (single + double positive events).**
IFNG and CD137	IFNG+ CD137+ All	***	
IFNG and CD40L	IFNG+ CD40L+ All		
TNF and CD137	TNF+ CD137+ All	**	
TNF and CD40L	TNF+ CD40L+ All		
CD137 and CD40L	CD137+ CD40L+ All	**	

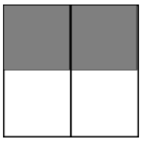

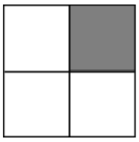

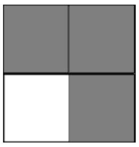

* Used for DR score calculation for the CD8+ T cell subset.
** Used for DR score calculation for the CD4+ T cell subset.
*** Used for DR score calculation for the CD8+ and CD4+ T cell subsets.
